# Do visually impaired children and their parents agree on the child's vision-related quality of life and functional vision?

**DOI:** 10.1136/bjophthalmol-2016-308582

**Published:** 2016-06-07

**Authors:** Valerija Tadić, Phillippa M Cumberland, Gillian Lewando-Hundt, Jugnoo S Rahi

**Affiliations:** 1 Population, Policy and Practice Programme, Life Course Epidemiology and Biostatistics Section, University College London (UCL) Institute of Child Health, London, UK; 2 Great Ormond Street Hospital for Children NHS Foundation Trust, London, UK; 3 Ulverscroft Vision Research Group, London, UK; 4 Warwick Medical School, University of Warwick, Coventry, UK; 5 National Institute for Health Research Biomedical Research Centre at Moorfields Eye Hospital NHS Foundation Trust and UCL Institute of Ophthalmology, London, UK

**Keywords:** Child health (paediatrics), Epidemiology

## Abstract

**Aims:**

To investigate agreement between children with visual impairment (VI) and their parents on their ratings of the child's vision-related quality of life (VQoL) and functional vision (FV) using two novel self-report patient-reported outcome measures developed for this population.

**Methods:**

99 children aged 10–15 years (mean age=12.2, SD=1.9) with VI (best corrected acuity (logarithm of the minimum angle of resolution) 0.50 or worse in better eye) and their parents participated in a national postal survey, completing the child and proxy versions of our novel instruments assessing VQoL and FV of children with VI—the vision-related quality of life instrument for children and young people (VQoL_CYP) and the functional vision questionnaire for children and young people (FVQ_CYP), respectively. Parent-child agreement was investigated using the Bland-Altman (BA) method. Variation across key sociodemographic and clinical characteristics was examined using the Intraclass Correlation Coefficient.

**Results:**

Average parental ratings of their child's VQoL and FV were significantly lower than the children's own ratings, but the range of disagreement was wide, with parents both overestimating and underestimating their child's VQoL (mean score difference=5.7, BA limits of agreement (LOA): lower −22.10 (CI 95% −24.61 to 19.59) and upper 33.50 (CI 95% 30.99 to 36.01)), but more consistently underestimating the child's FV (mean score difference=−11.8, BA LOA: lower −39.60 (CI 95% −42.12 to 37.08) and upper 16 (CI 95% 13.48 to 18.52)). There was variation in agreement by some child characteristics, including vision level, time of onset and course of VI progression.

**Conclusions:**

Visually impaired children and their parents perceive the broader impact of living with VI very differently. There is value in routine capture of information independently from children and their parents for comprehensively gauging the impact of childhood VI and tailoring appropriate interventions.

## Introduction

Visual impairment (VI) in childhood has significant far-reaching and lifelong impact with consequences for the child's social and educational experiences and future career prospects.^1–3^ Knowledge about children's own perceptions of the impact of living with VI, in terms of day-to-day functioning and quality of life (QoL) is limited, due to the paucity of vision-specific patient-reported outcome measures (PROMs) for this population.

Health-related QoL (HRQoL) is a complex construct shaped by personal lived experience and expectations in the context of a health condition,^4^ most accurately assessed by *self-reporting*, which can be by children as young as 5 years.^5^ Nevertheless, parents are still frequently asked to report as proxies on their child's HRQoL and functioning. However, an extensive literature shows that there is a high level of child-parent discordance on measures where both child self-report and parent proxy questionnaire versions are used.^6^ ^7^

Agreement between parental proxy and children's own reports of the impact of VI has only previously been examined in two studies,^8^ ^9^ both using the PedsQL,^10^ a generic HRQoL measure, in the absence, at the time, of a vision-specific measure. Generic measures do not capture vision-specific issues so the nature and the extent of child-parent discordance may not accurately represent the impact of the child's VI per se.

In the present study, we examined agreement between children with VI and their parents, and whether this varied by key clinical and sociodemographic child characteristics, using two novel self-report PROMs we recently developed specifically for this population. One assesses vision-related quality of life (VQoL)^11^ and the other functional vision (FV),^12^ each uniquely capturing the impact of living with VI in children.

## Method

The study was approved by the National Health Service Research Ethics Committee for University College London (UCL) Institute of Child Health and Great Ormond Street Hospital (GOSH), London, UK, and followed the tenets of the Declaration of Helsinki. The parents and children gave written consent and assent respectively to participation.

### Sample

The sample was drawn from (a) patient databases from the Department of Ophthalmology and the Developmental Vision Clinic at GOSH, and the Paediatric Glaucoma Service and Genetic Eye Disease Service at Moorfields Eye Hospital, London, UK and (b) 14 additional Paediatric Ophthalmology Departments UK wide (see Acknowledgements).

Children were eligible if (i) they were visually impaired or blind^i^ (corrected visual acuity (VA) in the better eye logarithm of the minimum angle of resolution (LogMAR) 0.50 or worse, using the WHO's definition of VI^13^ to capture all eligible children meeting this criteria regardless of severity) due to any visual disorder, but without any other impairment (ie, learning, sensory, motor) that would impact on their ability to self-report on or confound the specific impact of VI and if (ii) they were aged 10–15 years.

### Procedures

Eligible children and their parents were invited to participate in a postal survey evaluating the two novel vision-specific PROMs we were developing—the vision-related quality of life instrument for children and young people (VQoL_CYP)^11^ and the functional vision questionnaire for children and young people (FVQ_CYP).^12^ Each family received a study pack containing an invitation letter, information sheets for children and parents, consent and assent forms, large print and electronic (CD) versions of the child and parental instrument versions, described below, and a prepaid postage reply envelope.

The *VQoL_CYP*^11^ is a 35-item self-report questionnaire capturing the visually impaired child's perception of the impact of their visual disability in the societal context (from social relationships and psycho-emotional well-being to their autonomy and independence). The respondent child reports ‘how much they are like’ (child form) and the respondent parent ‘how much their child is like’ (parent form) the statement presented by each item (eg, ‘ feeling lonely because of my/her eyesight’), using a four-point scale (ranging from ‘1: not at all’ to ‘4: exactly’). The four response categories are converted to 0–3 scores (with negative items reversed) to derive a VQoL summary score, with higher summary scores indicating *better* VQoL (possible score range 0–105).

The *FVQ_CYP*^12^ is a 36-item self-report instrument assessing the visually impaired child's level of difficulty in performing activities for which vision is required. The respondent child or parent is asked to report the level of ‘ease’ with which the child performs the activity presented in each item (eg, ‘watching TV’) using a four-point scale (ranging from ‘very easy’ to ‘very difficult or impossible’). The four categorical responses are converted into 0–3 scores to derive a FVQ_CYP summary score, with higher summary scores indicating greater FV difficulty (possible score range 0–108).

### Data analysis

Summary scores on the two instruments were calculated for children and parents and score distribution screened for normality. Internal consistency was examined using Cronbach α coefficients.^14^ Paired-samples t tests were used to compare the means scores for children and parents.

Agreement between child and parent scores on the two instruments was assessed using the Bland-Altman method of limits of agreement (LOAs)^15^ and Intraclass Correlation Coefficients (ICCs). The variation in child-parent agreement was examined by children's sociodemographic factors (child age, gender, ethnicity and socioeconomic status using the Index of Multiple Deprivation based on the UK postal code^16^) and clinical characteristics (ie, VI level, progression and time of onset). To calculate the ICCs corresponding to these variables, in keeping with extant literature on child-parent agreement in paediatric HRQoL,^5^ a two-way mixed model (absolute agreement, single measure) was used, applying previously defined categories for the magnitude of agreement (≤0.40=poor to fair, 0.41–0.60=moderate, 0.61–0.80=good, 0.81≤excellent).^17^

Before calculating the summary scores, we carried out multiple regression-based imputation^18^ to replace the missing score data (threshold for missing data of <20% at item level and <25% at person level^12^). We report pooled mean score estimates across the multiple imputed datasets (five imputations). The Bland-Altman comparisons, t tests and ICCs were done across all the imputed datasets. As there were no significant variations between the results from different datasets, we report the estimates and plots using the first imputed data set only.^19^

Analyses were performed using SPSS (V.21.0).

## Results

### Participants

Ninety-nine families consented/assented to participation. Eighty-two per cent of parent responders were mothers and 85.4% from white ethnic majority backgrounds.

Table 1 shows clinical and sociodemographic characteristics of children and parents. The child participants were representative of the UK population of children with VI and blindness without additional impairments.^20^

**Table 1:** Clinical and sociodemographic characteristics of children

Child characteristics	N (%) total 99
Age group*	
10–12 years	61 (62%)
13–15 years	38 (38%)
Gender	
Boys	57 (58%)
Girls	42 (42%)
Ethnicity	
Majority ethnicity (white ethnic groups)	81 (81.8%)
Minority ethnicity (Asian, black, mixed, other non-white)	18 (18.2%)
Index of multiple deprivation†	
1: most deprived	20 (21.1%)
2	11 (11.6%)
3	19 (20%)
4	20 (21.1%)
5: least deprived	25 (26.3%)
*Vision level*‡	
VI group A	
VI 1: LogMAR 0.50–0.70	43 (43.4%)
VI 2: LogMAR 0.72–1.00	35 (35.4%)
*VI group B*	
SVI: LogMAR 1.02–1.30	10 (10.1%)
Blind: LogMAR 1.32 or worse	11 (11.1%)
Course of visual loss	
Stable§	55 (55.6%)
Progressive	44 (44.4%)
Timing of VI onset	
Early (≤2 years)	71 (71.7%)
Late	28 (28.3%)
Diagnosis by site of VI¶	
Whole globe and anterior segment	2 (2%)
Glaucoma—primary or secondary	8 (8.2%)
Cornea (sclerocornea and corneal opacities)	4 (4.1%)
Lens (cataract and aphakia)	10 (10.2%)
Uvea	6 (6.1%)
Retina	64 (65.3%)
Optic nerve	10 (10.2%)
Cerebral/visual pathways	5 (5.1%)
Other (idiopathic nystagmus, high refractive error)	11 (11.2%)

*Mean age=12.2, SD=1.9. †Based on UK postal code supplied by clinical team (missing in four children).

‡WHO categories of visual impairment based on acuity in better seeing eye. §Acceleration of visual loss was determined by the review of the notes and visual impairment characteristics by the leading author's (consultant ophthalmologist). ¶Does not add up to 100% because some children had visual impairment originating in multiple sites (missing in 1 child as diagnosis could not be obtained from the hospital where the patient was identified).

LogMAR, the logarithm of minimum angle of resolution; SVI, severe visual impairment; VI, visual impairment.

### Data screening

Of 99 consenting families, 90 child-parent pairs completed the VQoL_CYP and 93 the FVQ_CYP.

At item level, the amount of missing data for VQoL_CYP was ≤3% and for FVQ_CYP ≤16% (the reasons for missing data on the FVQ_CYP have been discussed elsewhere^12^). Data of four child-parent pairs had >25% missing data at the person level on FVQ_CYP, so were excluded from the subsequent analyses. The multiple regression-based imputation of missing data and summary score calculation was carried out for 90 and 89 child-parent pairs for VQoL_CYP and FVQ_CYP, respectively.

### Score distribution and reliability

The score distributions were within accepted normality limits (skewness between –1.0 and +1.0). Cronbach α coefficients for child and parent scores fell within the reliability criteria required for group and individual comparisons^21^ (children: 0.90 and 0.97 and parents: 0.92 and 0.95 on VQoL_CYP and FVQ_CYP respectively).

### Child-parent agreement

**Figure 1:**
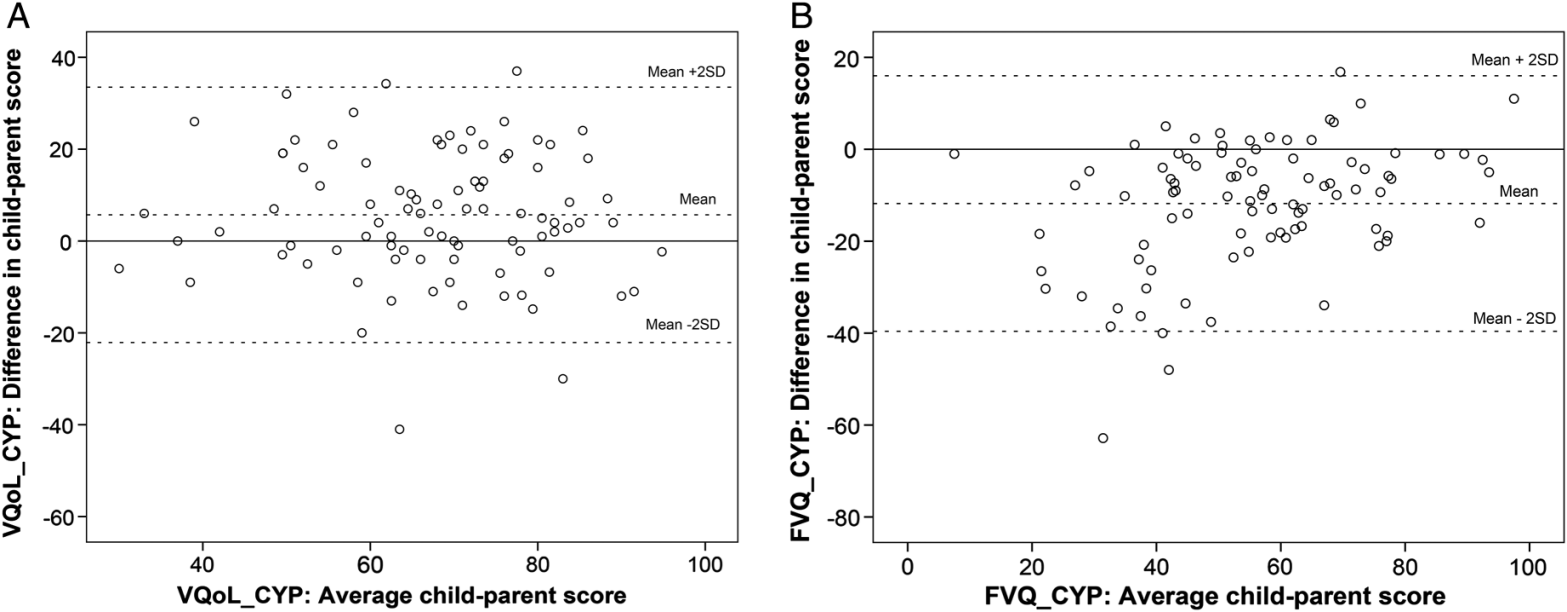
Bland-Altman plots of child-parent pair scores on vision-related quality of life instrument for children and young people (VQoL_CYP) (A) and functional vision questionnaire for children and young people (FVQ_CYP) (B). On the VQoL_CYP, higher scores indicate better vision-related quality of life outcome, whereas on the FVQ_CYP, higher scores indicate greater functional vision difficulty.

**Figure 2:**
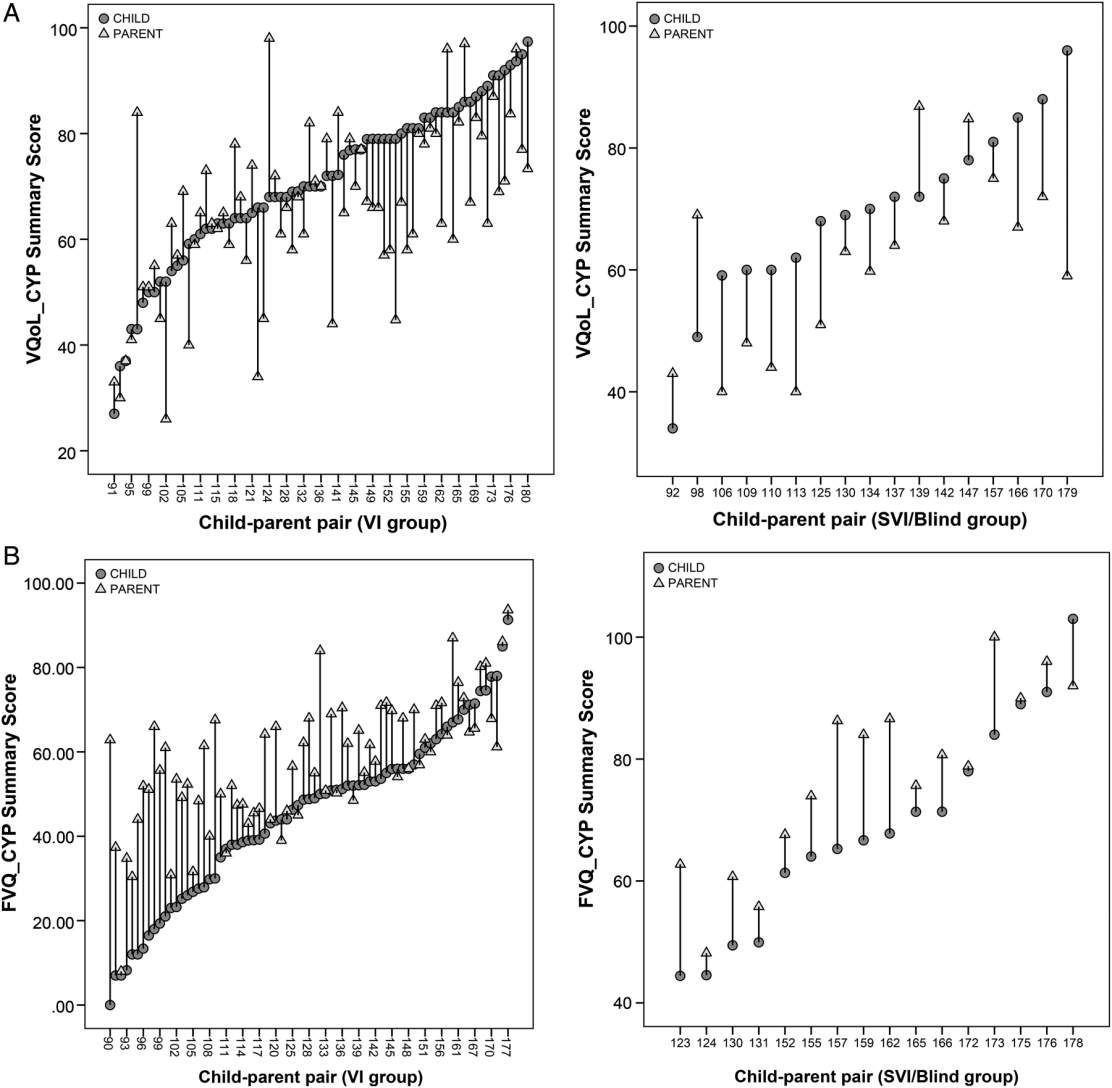
Discrepancy in child-parent scores on vision-related quality of life instrument for children and young people (VQoL_CYP) (A) and functional vision questionnaire for children and young people (FVQ_CYP) (B) in individual pairs by visual impairment level. On the VQoL_CYP, higher scores indicate better vision-related quality of life outcome, whereas on the FVQ_CYP, higher scores indicate greater functional vision difficulty. Visual impairment (VI): visual acuity logarithm of the minimum angle of resolution (LogMAR) in better eye=0.50–1.00; severe VI (SVI)/blind: LogMAR worse than 1.00.

On average, parents rated their children as having significantly poorer VQoL and FV than did children themselves (paired t tests: p<0.001) (table 2). However, the range of child-parent disagreement was wide and in both directions (table 2, figure 1). While parents tended to both underestimate and overestimate their child's VQoL, they consistently underestimated their child's FV ability. This directional pattern of discrepancy appeared consistent across the key clinical (eg, vision level, figure 2) and sociodemographic variables. Furthermore, greater child-parent discrepancy was observed where the parents underestimated rather than overestimated their child (figure 2), the pattern being particularly prominent in children with VI who rated themselves as having better FV (ie, lower scores) (figure 2, section B).

**Table 2:** Bland-Altman and ICC agreement between **c**hild-parent pairs on VQoL_CYP and FVQ_CYP summary scores

	Child summary score—mean (SD)	Parent summary score—mean (SD)	Mean paired score difference (SD) (95% CI)	Minimum difference	Maximum difference	Bland-Altman lower limit of agreement (CI 95%)	Bland-Altman upper limit of agreement (CI 95%)	ICC (95% CI)
VQoL_CYP*	70.5 (15.1)	64.8 (15.7)	5.7 (SD 13.9)† (2.8 to 8.6)	−41	37	−22.10 (−24.61 to −19.59)	33.50 (30.99 to 36.01)	0.56 - moderate agreement (0.37 to 0.7)
FVQ_CYP*	49.4 (21.7)	61.2 (16.8)	−11.8 (SD 13.9)† (−14.8 to −8.9)	−62.9	16.8	−39.60 (−42.12 to −37.08)	16 (13.48 to 18.52)	0.63 - good agreement (0.16 to 0.82)

*On VQoL_CYP, higher scores indicate better vision-related quality of life outcome, whereas on the FVQ_CYP higher scores indicate greater functional vision difficulty.

†Paired t test difference significant at p<0.001. FVQ_CYP functional vision questionnaire for children and young people; ICC, Intraclass Correlation Coefficient; VQoL_CYP, vision-related quality of life instrument for children and young people.

ICCs in table 3 show the variation in magnitude of child-parent agreement by clinical and sociodemographic variables by agreement categories, with average level agreement ranging from ‘moderate’ to ‘good’ across the two measures.

**Table 3:** Variation in mean child and parent VQoL and FV scores by clinical and sociodemographic characteristics

	VQoL_CYP*			FVQ_CYP*		
	Mean		ICC (CI 95%)	Mean		ICC (CI 95%)
	Child	Parent		Child	Parent	
Age						
10–12 years	71.2	65.5	0.58 (0.355 to 0.738)	51.2	62.9	0.63 (0.219 to 0.839)
13–15 years	69.5	63.6	0.52 (0.229 to 0.729)	46.5	57.9	0.61 (0.170 to 0.821)
Gender						
Girls	70.2	65.2	*0.48 (0.195 to 0.689)†*	55	64.9	0.61 (0.195 to 0.813)
Boys	70.8	64.6	*0.62* (*0.366 to 0.773)*	45.3	58.2	0.62 (0.095 to 0.828)
Vision level						
VI (LogMAR 0.50–1.00)	70.8	65.8	0.58 (0.386 to 0.719)	45.2	57.6	*0.53 (0.091 to 0.752)*
SVI/blind (LogMAR worse than 1.00)	69.3	60.8	0.47 (0.031 to 0.763)	68.9	76.9	*0.76* (*0.115 to 0.928)*
Timing of VI onset						
Early (≤2 years)	71.8	64.8	0.55 (0.296 to 0.723)	47.6	60.3	*0.54* (*0.085 to 0.766)*
Late	67.6	64.8	0.57 (0.259 to 0.779)	54	62.8	*0.80* (*0.344 to 0.926)*
Course of visual loss						
Stable	71.9	65.1	0.52 (0.248 to 0.707)	46.2	60	*0.53 (−0.011 to 0.785)*
Progressive	68.8	64.5	0.60 (0.366 to 0.769)	53.4	62.3	*0.72 (0.383 to 0.862)*
Child ethnicity						
White British majority	70.5	64.8	*0.53 (0.321 to 0.683)*	48.3	59.6	0.62 (0.168 to 0.809)
Other UK minority	71	64.8	*0.67 (0.281 to 0.875)*	55.8	68.7	0.65 (0.049 to 0.888)
Deprivation (UK population quintiles)						
1, 2, 3 more deprived	68.8	63.6	*0.65* (*0.420 to 0.801)*	52.3	64.9	*0.55* (*0.037 to 0.788)*
4, 5 least deprived	73.5	66.6	*0.51* (*0.220 to 0.708)*	45.8	56.8	*0.66* (*0.227 to 0.838)*

*On VQoL_CYP, higher scores indicate better vision-related quality of life outcome, whereas on the FVQ_CYP, higher scores indicate greater functional vision difficulty.

†The results in italics show variation in agreement by different agreement categories (≤0.40=poor to fair, 0.41–0.60=moderate, 0.61–0.80=good, 0.81≤excellent) by group.

FV, functional vision; FVQ_CYP, functional vision questionnaire for children and young people; ICC, Intraclass Correlation Coefficients; LogMAR, logarithm of minimum angle of resolution; SVI, severe visual impairment; VI, visual impairment; VQoL_CYP, vision-related quality of life instrument for children and young people.

There were some notable differences in agreement categories for some characteristics on FVQ_CYP, that is, visually impaired children: ‘moderate’, severely visually impaired or blind children: ‘good’; early VI onset: ‘moderate’, late VI onset: ‘good’; stable VI: ‘moderate’, progressive VI: ‘good’; more deprived socioeconomic background: ‘moderate’, least deprived background: ‘good’. Equally, such differences were noted also for the VQoL_CYP, that is, girls: ‘moderate’, boys: ‘good’; white British majority ethnic background: ‘moderate’, other ethnic minorities: ‘good’; more deprived: ‘good’, least deprived: ‘moderate’.

Additionally, based on the agreement categories, potentially higher (ie, ‘good’ vs ‘moderate’) agreement was observed on FVQ_CYP than on VQoL_CYP on some child characteristics (eg, girls and late and more progressive visual loss).

## Discussion

The present study investigated concordance between child self-report and parental proxy report on the impact of the child's VI on his/her VQoL and FV, using novel vision-specific PROMs for children with VI.

We found that visually impaired children's and their parents' perspectives of the impact of VI on the child differed significantly. Parents on average perceived their child's VI as having a greater impact on the child compared with their child's own rating. The range of child-parent disagreement was wide, with parents both underestimating and overestimating their child's own report. The extent of child-parent discrepancy varied by certain clinical and sociodemographic characteristics of the children as well as by instrument. The pattern of parental underestimation was particularly prominent for visually impaired children who rated their outcomes more favourably.

Patterns of agreement/discordance in this study are similar to those in other studies on child-parent agreement on child's health outcomes in other non-VI paediatric populations.^6^ ^7^ They also extend the findings of prior studies examining agreement between children with VI and their parents using a generic HRQoL measure.^8^ ^9^

Our study design and limited variable information precluded the opportunity to assess other variables potentially influencing discordance, such as parental health and well-being, parental age and educational level, and number of siblings,^6^ ^22^ ^23^ which we will address in future studies with larger samples. We did not find greater discordance with increasing age of child, as anticipated, which probably reflects the narrow age range of our sample compared with other studies.^6^ ^24^ ^25^ Study resources necessitated a postal survey, preventing ascertaining the level of parental help received by children and the extent to which this may have affected informant agreement. The size of our sample, reflecting the vulnerable, heterogeneous and numerically small clinical population of children with VI, precluded us from investigating the nature of and variation in informant discrepancy in greater detail. However, the variation observed is generalisable as our sample is representative of the UK population of children with VI without additional impairments.^20^ A limitation of the sample size is that the differences in variation by child characteristics did not reach statistical significance. The variation by gender, ethnicity and socioeconomic status is interesting, although complex to interpret given the conflicting wider literature in this area.^26^

The nature and extent of child-parent discordance is likely to vary by the type and severity of the child's condition, the domain or construct being measured, the child's age, gender, type of condition, duration of illness and treatment status.^6^ Greater agreement is typically found on more observable, physical characteristics and greater divergence on unobservable emotional and psychosocial characteristics of the impact of the health conditions.^7^ ^24^ We found greater magnitude of agreement, by agreement categories, on FVQ_CYP than on VQoL CYP, irrespective of the direction of disagreement. The FVQ_CYP was designed to capture the difficulty with which a child performs vision-dependent activities (eg, the level of difficulty with which a child navigates around the school or finds friends in the playground) and thus may be objectively more agreement prone than the psychological characteristics that the VQoL_CYP was intended to capture (eg, autonomy, social inclusion, emotional well-being).

Our finding of potentially greater child-parent agreement, especially on FVQ_CYP, in children with progressive VI echoes findings relating to systemic diseases where, arguably, active illness demands greater child-parent communication and parental vigilance about symptoms and illness characteristics than in non-progressive disease, thus resulting in greater child-parent agreement.^6^ ^27^ We also found a potentially greater agreement on FVQ_CYP for children with more severe and late onset VI, both of which tend to coincide with progressive loss of vision. Parents of children with progressive, late onset, visually impairing disorders, such as Stargardt disease, may be more in tune with their child's rapid and/or fluctuating loss of function as their child may become increasingly dependent on parental help and support (especially relating to functional outcomes), which in turn may result in greater child-parent communication and ultimately agreement. These findings may have potentially important clinical implications in the scenario of distress and depression in teenagers with rapid loss of vision and function; knowing the child-parent agreement is higher for this group may be helpful in the clinical monitoring of and research with children who may be too distressed and thus potentially unable to self-report themselves at particular stages.

The reasons for the child-parent disagreement are not fully understood, but there are several possible explanations. For instance, parents of children with VI may underestimate their child's FV because they may focus on a bigger ‘life’ picture and weight their perceptions of their child's visual ability against their own worries and concerns, a particular life demand (eg, independent living), other children's abilities and the general implications for the future. Conversely, children, particularly when younger, may focus on their current level of functioning rather than making comparisons with others. With respect to the VQoL_CYP, the bidirectional pattern of child-parent discordance may be down to the parental reports likely being influenced by the degree to which parents can observe different settings (eg, their child's social lives at school) that are likely to influence on how children feel on a daily basis.^6^ Finally, the general reasons for disagreement could be methodological as children and parents use different response styles in completing questionnaires whereby children are more likely to provide extreme scores as well as provide different explanations for choosing those response options.^28^

We are currently adapting our novel PROMs of VQoL and FV to younger and older patients, which will enable the investigation of potential age-related differences. The planned use of these PROMs in routine clinical practice planned in our clinical centres will enable us to evaluate the nature and extent of and need for parental assistance in completing these questionnaires. This future planned work will also enable us to establish with more accuracy a clinically minimally important difference for individual children's scores over time and therefore the clinically significant meaning of the child-parent difference in scores for different measures.

In summary, we report findings on child-parent concordance and divergence in evaluation of the child's VQoL and FV using vision-specific instruments. There is a wide range of disagreement in how visually impaired children and their parents perceive the functional as well as psychological impact of living with VI on the affected child's life, which to an extent is influenced by the child's clinical or sociodemographic characteristics. Clinicians should not disregard this discordance, which is likely to be highly informative for the purpose of clinical management of individual patients, especially in older children. The information provided by children and their parents should be viewed as being complementary, rather than interchangeable. Our findings highlight a potential value in *routinely* capturing both perspectives for their unique contribution in comprehensively gauging the impact of childhood VI and tailoring appropriate interventions, both in clinical practice and research.

## Footnotes

i For brevity, we consider term visual impairment (VI) in the remainder of the paper.
